# Supplementary material for: The causal relationship between the human gut microbiota and pyogenic arthritis: a Mendelian randomization study
Source: Front Cell Infect Microbiol. 2024 Nov 26;14:1452480. doi: 10.3389/fcimb.2024.1452480 (PMC11629706; doi:10.3389/fcimb.2024.1452480)
Supplement: Supplementary file 1 [file DataSheet1.zip › mendelian test/ebi-a-GCST90027857.csv_forest.pdf]

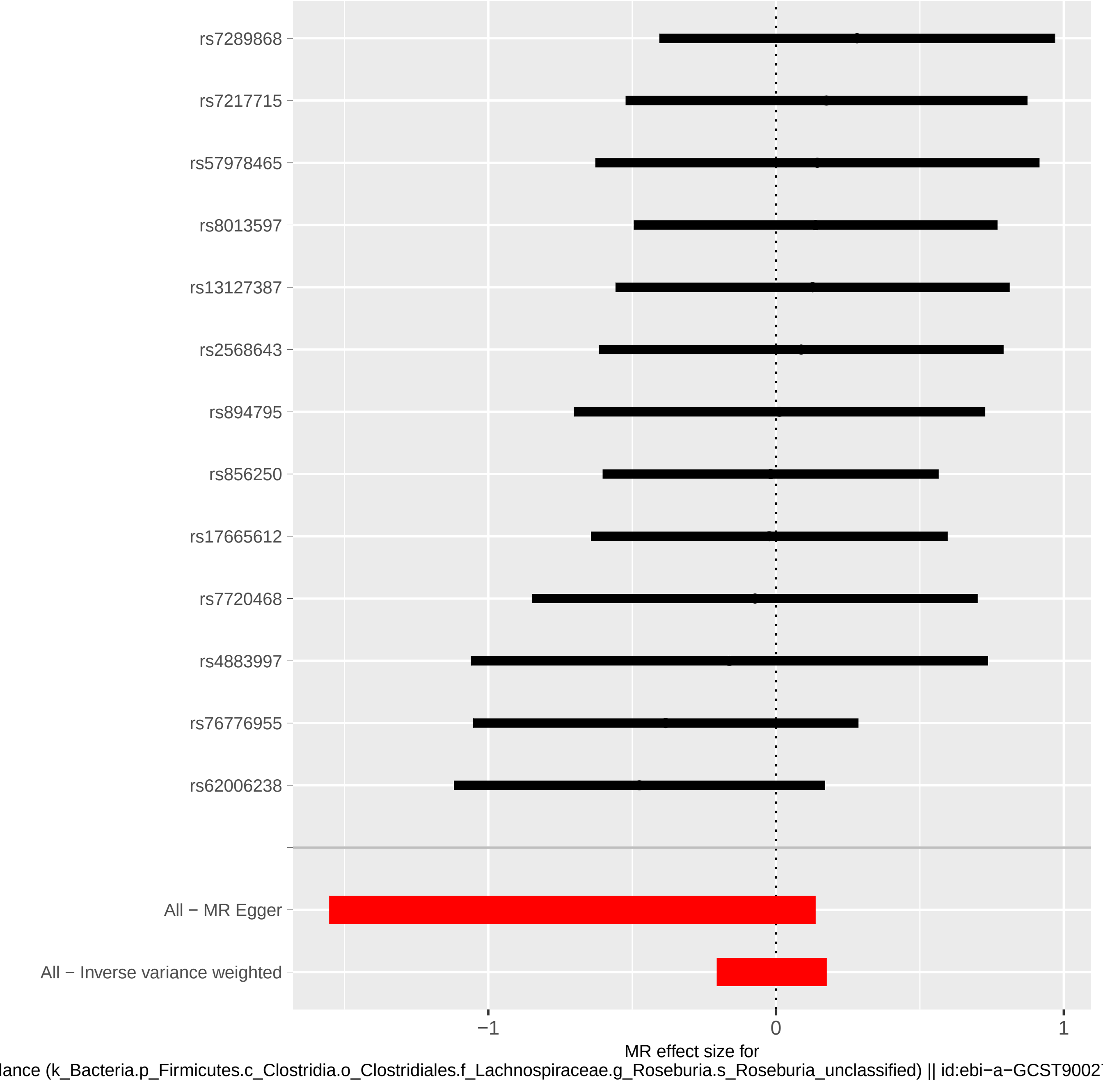

rs7289868
rs7217715
rs57978465
rs8013597
rs13127387
rs2568643
rs894795
rs856250
rs17665612
rs7720468
rs4883997
rs76776955
rs62006238
All − MR Egger
All − Inverse variance weighted
−1
0
1
MR effect size for
lance (k_Bacteria.p_Firmicutes.c_Clostridia.o_Clostridiales.f_Lachnospiraceae.g_Roseburia.s_Roseburia_unclassified) || id:ebi−a−GCST9002
